# Fracture Statistics for Inorganically-Bound Core Materials

**DOI:** 10.3390/ma11112306

**Published:** 2018-11-16

**Authors:** Philipp Lechner, Jens Stahl, Florian Ettemeyer, Benjamin Himmel, Bianca Tananau-Blumenschein, Wolfram Volk

**Affiliations:** 1 Chair of Metal Forming and Casting, Technical University of Munich, Walther-Meissner-Strasse 4, 85748 Garching, Germany; jens.stahl@utg.de (J.S.); benjamin.himmel@utg.de (B.H.); btananau@web.de (B.T.-B.); wolfram.volk@utg.de (W.V.); 2 Fraunhofer Research Institution for Casting, Composite and Processing Technology IGCV, Walther-Meissner-Strasse 4, 85748 Garching, Germany; Florian.ettemeyer@igcv.fraunhofer.de

**Keywords:** inorganic sand core materials, Weibull, fracture strength, water-glass, three-point-bending, four-point-bending

## Abstract

In this article, we study the fracture characteristics of inorganically-bound foundry cores. It will be shown that the fracture stress of inorganic cores follows Weibull’s strength distribution function for brittle materials. Using three-point and four-point-bending experiments, the volume dependence of the bending fracture stress is analyzed and a Weibull model fitted. Furthermore, the fracture stress of arbitrary bending experiments can be calculated based on the Weibull parameters found.

## 1. Introduction

### 1.1. Mechanical De-Coring Properties of Inorganically-Bound Sand

Inorganically-bound sand is used in the casting industry as a mold material. The sand particles are mixed with an inorganic binder system, in most cases water-glass, and hardened with a condensation reaction induced by heat [1].

The resulting porous material is temperature-tolerant enough to serve as a material for casting molds and cores in light metal foundries [2]. Inorganically-bound core materials are more ecofriendly than organically-bound core materials. Due to the absence of carbon compounds in the binder system, no combustion products emerge, except for water steam [3]. This leads to a higher residual strength of the cores after casting compared to the organically-bound ones, whose binder is partially destroyed by combustion [1].

The sand binder mixture is shaped into sand cores by core blowing machines. The resulting cores are used for geometries in the casting part, which cannot be demolded. After casting, the sand cores have to be de-agglomerated until the remaining parts can be removed from their respective cavity, which is called “de-coring”. De-agglomeration can be achieved by various techniques, such as hammering and shaking [4], or with pressurised air [5], or shock waves [6]. All of these de-coring techniques represent a strain, not only on the core, but also for the cast part.

The dimensioning and parameters of the mechanical de-coring process are mostly based on experience. Due to ever-more complex casting geometries, thinner wall thicknesses and new developments in the field of molding materials, an early prediction of de-coring properties will become necessary. Furthermore, this might offer the possibility to optimize the cores’ properties in order to reduce the de-coring effort. For this purpose, knowledge of the mechanical fracture behavior of the cores themselves is an essential prerequisite. Much work has been done on the bending strength of sand core materials. Stauder et al. measured the mechanical properties of sand cores by three-point-bending [7]. Furthermore, Griebel et al. analyzed strain on the surface of four-point-bending beams with optical measurement techniques [8]. Izdebska-Szanda and Balinski studied the influence of heat treatment on the strength of inorganic sand cores [9]. However, no published work can be found on the fracture statistics and the influence of the test specimens’ stress state and geometry on the resulting strength. Therefore, this article is concerned with the fracture statistics of inorganically-bound sand cores.

### 1.2. Weibull Statistics

The Weibull statistics is a probability distribution function. It is widely applied in the fracture statistics of brittle materials such as ceramics, because they typically follow the weakest link theory. Brittle fractures occur with little to no plastic deformation. The fracture originates where the weakest (major) defect is located and stresses are concentrated in brittle materials. Therefore, the strength of the major defect affects the individual material strength [10].

During the tests, the load is constantly increased until the tensile stress at the critical defect reaches a value that is higher than the local strength. With brittle materials, the material strength is described by means of a strength distribution function measured from a set of test specimens that are identical and tested under the same conditions. The Weibull statistics show the probability of failure while considering different aspects such as the state of stress or the stress amplitude. Using Weibull’s theory, the number of specimens, which are fractured to measure the strength distribution, is usually around 30 [10].

In this article, the experiments are plotted using logarithmic scales, with the induced stress in the *x*-direction and the fracture probability *F* in the *y*-direction. Additionally the value for $lnln\frac{1}{1-F}$ is added, which leads to one of the Weibull parameters ($\sigma_{s}$) at zero.

In theory, a perfectly Weibull-distributed set of data points should lead to a straight line in this plot. The Weibull distribution used is described by two parameters, $\sigma_{s}$ and *m*. $\sigma_{s}$ is called the scale parameter, which describes the stress causing 63.2% of the specimen to fracture. *m* is called the shape parameter, because it describes the slope of the Weibullian line. *m* is considered a material constant, while $\sigma_{s}$ shifts depending on the stress applied [11].

The probability of failure in a pure tensile test with constant volume and stress is described as follows by Weibullian statistics [12]:

(1) $$P_{f}\left(\sigma \right)=1-\exp\left\{-{\left[\frac{\sigma }{\sigma_{s}}\right]}^{m}\right\}$$

where $P_{f}$ is the probability of failure and σ is the applied stress. Comparing specimens with different volumes *V* or stress, $\sigma_{s}$ follows [10]:

(2) $$\frac{V_{1}}{V_{2}}={\left(\frac{\sigma_{s_{2}}}{\sigma_{s_{1}}}\right)}^{m}$$

In order to calculate the probability of failure based on the bending strength $\sigma_{b}$ of bending tests, the effective volume $V_{eff}$ has to be calculated, since the stress in the specimen’s volume is not constant [13]:

(3) $$V_{eff}=\int_{V}{\left(\frac{\sigma }{\sigma_{b}}\right)}^{m}dV$$

where σ is the local stress in the volume cell used for the integration and $\sigma_{b}$ is the bending strength of the specimen.

## 2. Materials and Methods

In order to describe the fracture behavior properly, various test setups such as tensile, bending or pressure tests are available. In this study, bending tests were chosen, since they are easy to perform and the induced stresses can be evaluated analytically using the beam theory. This assumes ideal Bernoulli beams with infinitesimally small load and support points, as well as homogeneous material properties. The validity of this simplification will be checked in Section 4.

Here, only the tensile component of the bending stress is considered for further calculations since the compressive strength is assumed much higher than the tensile strength for sand core materials.

### 2.1. Specimen

The sand core specimens used were produced using H32 quartz sand from Quarzwerke GmbH (Frechen, Germany) and W65 Bauxite sand from Hüttenes-Albertus Chemische Werke GmbH (Duesseldorf, Germany). Both sand types are bound with two inorganic Inotec binder systems from ASK Chemicals GmbH (Hilden, Germany). The binder system is composed of a liquid component and an additive in powder form and is measured in wt% relative to the sand mass. This system will be referred to as “binder” in the following sections. For Specimen Type A, H32 sand and 2 wt% fluid binder EP4158 with 1.9 wt% additive WJ4500 were used. For Specimen Type B, H32 sand and 2 wt% fluid binder EP4158 with 1.6 wt% additive TC4500 were used. For Specimen Type C, W65 sand and 2 wt% fluid binder EP4158 with 1.6 wt% additive TC4500 were used.

The specimens were produced on a Loramendi SLC2 25L core blowing machine (Loramendi S.Coop., Vitoria-Gasteiz, Spain) with a heated core box and a hot air drying device. The temperatures were set to 155 ${}^{\circ }$C core box and 220 ${}^{\circ }$C air temperature. After production, the specimens were stored for 20 h in a climate chamber at 20 ${}^{\circ }$C and 20% relative humidity. The dimensions of the sand cores were 22.8 × 22.8 × 170 mm^3^.

### 2.2. Uni-Axial Constant Stress

The mechanical testing was performed on a Zwick&Roell Z020 Universal Testing Machine (ZwickRoell GmbH & Co. KG, Ulm, Germany) with a 4-point-bending (4PB) and a 3-point-bending (3PB) fixture. The testing machine was equipped with a 20-kN load cell.

**Figure 1:**
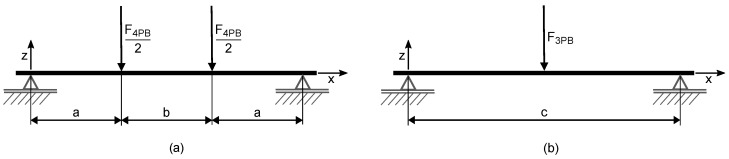
Test setup for the 4PB (**a**) and 3PB (**b**) experiments.

In order to induce uni-axial constant stresses in the surface layer of the tensile side, 4-point-bending tests were performed. A specimen rests on the two lower supports and is strained with a flexural load through the upper supports. Figure 1a shows the test setup. This design ensures a constant and uni-axial stress between the upper supports in the *x*-direction for a given distance from the neutral layer and a linear increasing absolute stress in the *z*-direction. The distance between the supports *c* was chosen to be 150 mm and the testing speed as 1 mm/min. The distance between the load points was varied and will be listed with the results. The force-deflection value pairs were measured by the testing machine. Of these, only the maximum force was used to calculate the tensile stress component in the specimen according to beam theory with Föppl notation [14,15]:

(4) $$\sigma_{4PB}\left(x,z\right)=\frac{\frac{F_{4PB}}{2}\left(x-{\langle x-a\rangle }^{1}-{\langle x-\left(a+b\right)\rangle }^{1}\right)}{I}z$$

where $\sigma_{4PB}$ is the stress in the beam, depending on the coordinates *x* and *z* resulting from 4-point-bending, $F_{4PB}$ is the force applied by the testing machine, *a* is the distance from the supports to the load point and *b* is the distance between the load points. *I* is the geometrical moment of inertia, and *z* is the distance from the neutral layer.

### 2.3. Uni-Axial Non-Constant Stress

In order to induce uni-axial non-constant stress in the beam, 3-point-bending experiments were performed on the same Z020 testing machine. The test setup is depicted in Figure 1b. Again, the chosen distance between the supports was 150 mm. The stress in the the beam was determined according to [14,15]:

(5) $$\sigma_{3PB}\left(x,z\right)=\frac{\frac{F_{3PB}}{2}\left(x-2{\langle x-\frac{c}{2}\rangle }^{1}\right)}{I}z$$

where $\sigma_{3PB}$ is the stress in the beam depending on *x* and *z* and resulting from 3-point-bending, $F_{3PB}$ is the force applied by the testing machine and *c* is the distance between the supports. *I* is the geometrical moment of inertia, and *z* is the distance from the neutral layer.

### 2.4. Finite Element Model

**Figure 2:**
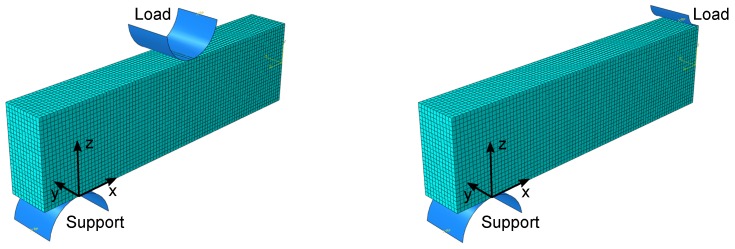
Quarter model of the beam for 4PB 75.5 mm and 3PB.

Deviations of the stress in the specimen from the Bernoulli equation are investigated by the finite element method (FEM) in Abaqus (Dassault Systems, Velizy-Villacoublay, France). A quarter of the beam was modeled using both symmetries, as displayed in Figure 2. A Young’s modulus of 7550 MPa and a Poisson’s ratio of 0.17 as published by Schneider et al. [16] were utilized. C3D8 elements (size: 1.00 × 1.04 × 0.99 mm^3^) were used for the beam, while the support and load were modeled by analytical rigid bodies with the same dimensions as measured in the universal testing machine. The beam was loaded with the force at a failure probability of 63.2%, corresponding to each load case.

## 3. Results

### 3.1. Characterisation of the Fracture Behavior

**Figure 3:**
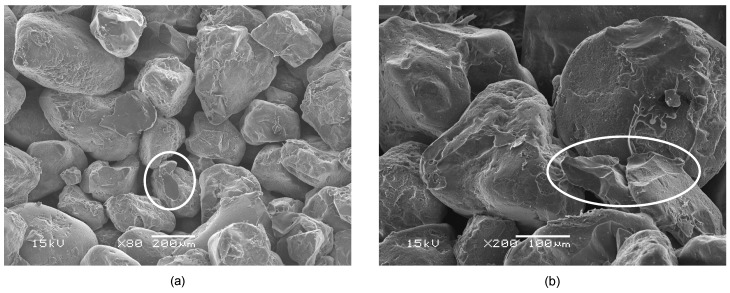
Overview of inorganically-bound quartz sand with broken binder bridges (**a**) and a broken binder bridge with signs of brittle failure (**b**).

In order to determine whether the fracture behavior can be described as brittle or ductile, SEM images of broken binder bridges were taken. Figure 3 shows broken binder bridges and an overview of the broken micro-structure in the sand-binder-compound. The SEM (Jeol, Tokyo, Japan) images show very sharp edges in the area of the broken water-glass binder bridges between the quartz particles, which indicates a brittle fracture.

The classical way to measure the strength of foundry cores is to use three-point-bending devices and breaking a statistically-relevant number of specimens. Evaluating these measured data points, the strength is then given as a mean value. Mostly, the scatter of the data points is neglected, or if studied, the standard deviation is given [7,8].

This implies that the fracture behavior follows the Gaussian probability distribution. However, inorganically-bound sand cores show a quite brittle material behavior. In materials science, this kind of fracture is described with Weibull’s probability distribution, not with the Gaussian one [17,18]. Consequently, in this article, experimental results are analyzed in relation to the probabilistic distribution of the specimens’ strength.

### 3.2. Probabilistic Distribution of the Strength of Inorganically-Bound Sand Cores

In this section, experimental results for the strength of inorganically-bound sand core materials will be shown and tested if the results can be described with a Weibullian probability distribution.

In order to derive the probability of failure for a specific dataset, which consists of fracture stress results for multiple specimens, the data need to be sorted by value in ascending order. The probability then follows [19]:

(6) $$P_{f}\left(\sigma_{i}\right)=\frac{i-0.5}{n}$$

where $P_{f}$ is the probability of failure, $\sigma_{i}$ is the *i*-th value in the sorted dataset and *i* is the position of this value in it. *n* is total number of measured points.

**Figure 4:**
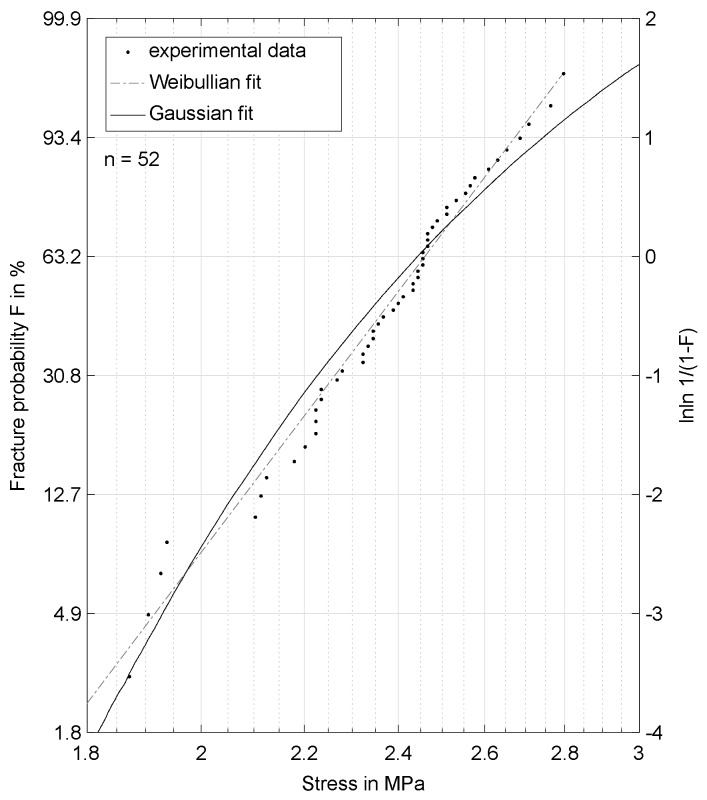
Strength probability distribution with Gaussian and Weibullian fit.

Figure 4 shows a Weibull plot of 52 inorganically-bound specimens. Additionally, Weibull and Gauss distributions were fitted to the data with the maximum likelihood method, utilizing MATLAB functions “wblfit” and “normfit” [20]. The data are plotted with logarithmic scales, which leads to a straight line if the data follow the Weibull distribution and a curved line if they follow the Gauss distribution. It is evident that the Weibullian distribution fits the data better than the Gaussian. To validate this assumption, a Lilliefors test was performed with MATLAB [20]. The hypothesis of a Gauss distribution was rejected at a 5.5% significance level, while the Weibull distribution was not rejected. Thus, a Weibullian distribution is assumed for all modeling purposes in this paper. A direct consequence of the Weibullian distribution is the volume-dependence of the specimens’ tensile and bending strengths. This can be easily shown with a four-point-bending and a three-point-bending fixture, since the effective volume $V_{eff}$ varies for different test setups. The two parameters of the Weibull distribution $\sigma_{s}$ and *m* can be calculated with Equations (1)–(3).

### 3.3. Volume-Dependence of the Tensile Strength

**Figure 5:**
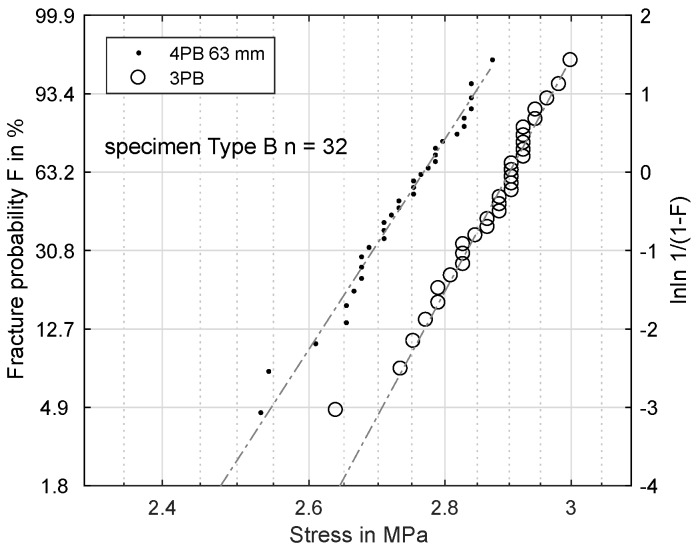
Fracture probability for a 3PB and a 4PB (load distance 63 mm) dataset with different effective volumes.

Figure 5 shows two sets of data. While the distance between the supports was constant, the effective volumes differed between the three-point-bending and the four-point-bending experiment according to Equation (3). The two datasets confirm the assumption of a volume-dependence: the bending strength was evidently influenced by the loaded effective volume, since the two datasets were clearly distinguishable and had different values for $\sigma_{s}$.

### 3.4. Predicting Fracture Stresses for Arbitrary Bending Test Setups

Using Equation (2), it is possible to calculate the scaling parameter $\sigma_{s}$ for arbitrary bending test setups based on one dataset. In order to compare the effective volumes of the three- and four-point-bending test setups, the whole volume of the four-point-bending beam is considered, not just the volume between the load points.

**Figure 6:**
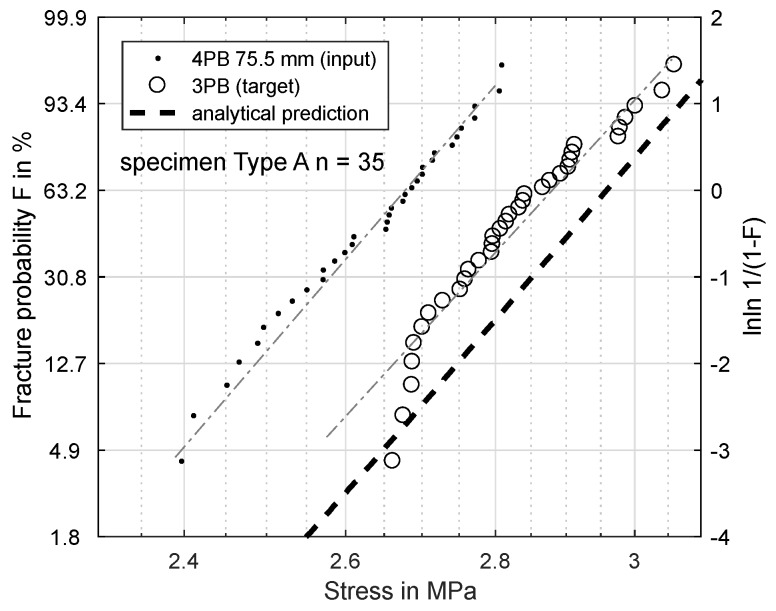
Specimen Type A: prediction of fracture probability from 4PB (75.5 mm) to 3PB.

**Figure 7:**
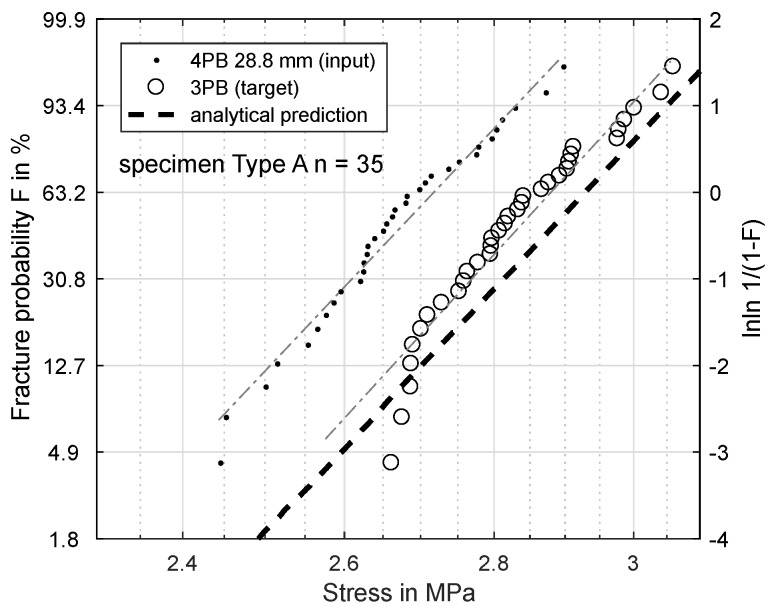
Specimen Type A: prediction of fracture probability from 4PB (28.8 mm) to 3PB.

**Figure 8:**
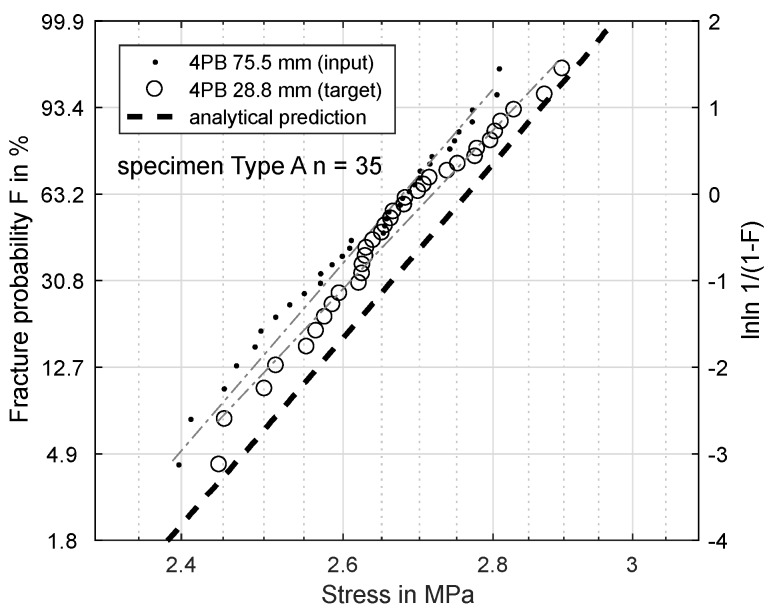
Specimen Type A: prediction of fracture probability from 4PB (75.5 mm) to 4PB (28.8 mm).

**Figure 9:**
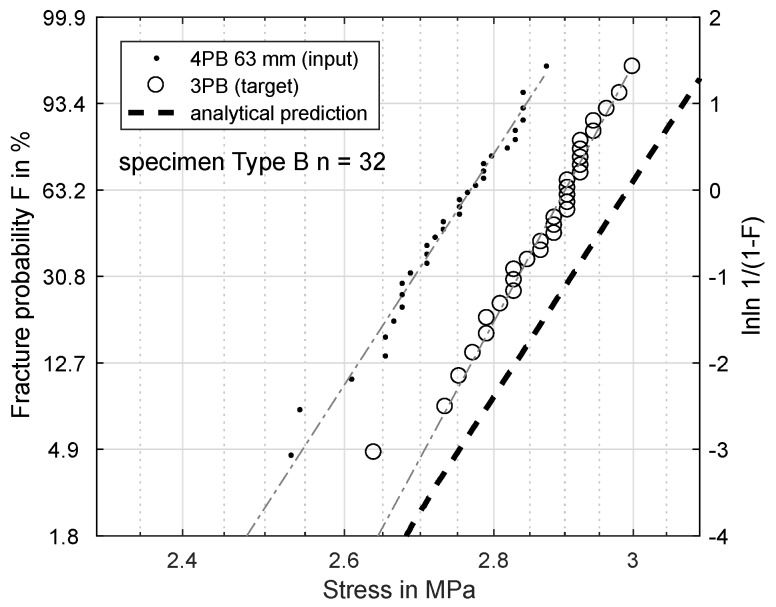
Specimen Type B: prediction of fracture probability from 4PB (63 mm) to 3PB.

Figure 6, Figure 7, Figure 8 and Figure 9 show the results of this prediction. They are structured as follows: The measured input dataset, upon which the prediction is based, is shown as points, the target data-set with circles and the prediction itself with a dashed line.

Figure 6 shows the prediction of a three-point-bending test based on a four-point-bending test with 75.5 mm load distance for specimens of Type A. The measured $\sigma_{s}$ is 2.880 MPa, and the predicted $\sigma_{s}$ is 2.959 MPa.

Figure 7 shows the same prediction based on a load distance of 28.8 mm. The predicted $\sigma_{s}$ is 2.929 MPa, which fits the experiment significantly better, though it still overestimats the bending strength by 0.05 MPa.

Figure 8 compares two 4PB tests. The prediction is calculated for the 28.8-mm load distance based on the 75.5-mm measurement. The prediction also overestimates the strength of the target (measured: 2.717 MPa, predicted: 2.763 MPa).

Clearly, the Weibull model overestimates the strength for smaller effective volumes to some degree. The effective volume in the four-point-experiment is inversely proportional to its load distance. The theoretical extreme is a load distance of zero with the same effective volume as for the three-point-bending. The closer the four-point load distance approaches this extreme, the more accurate the prediction becomes.

For validation reasons, a second type of specimen (B) was used for another four-point-/three-point-bending comparison. The load distance chosen was 63 mm, which is shown in Figure 9. As expected, the bending strength is over-estimated, and the offset of the prediction is similar to the 75.5-mm load distance of Specimen Type A (measured: 2.901 MPa, predicted: 2.992 MPa).

### 3.5. FEM Simulation of the Four-Point Bending Test Setup

**Figure 10:**
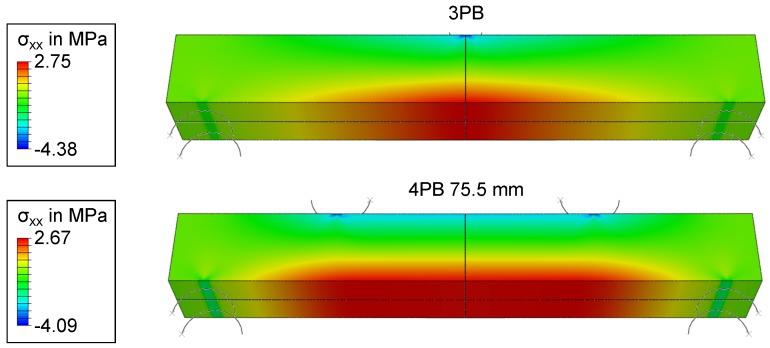
The FEM results show the stress in the *x*-direction of 3PB (top) and 4PB 75.5 mm (bottom), which were used for the calculation of the effective volume.

**Figure 11:**
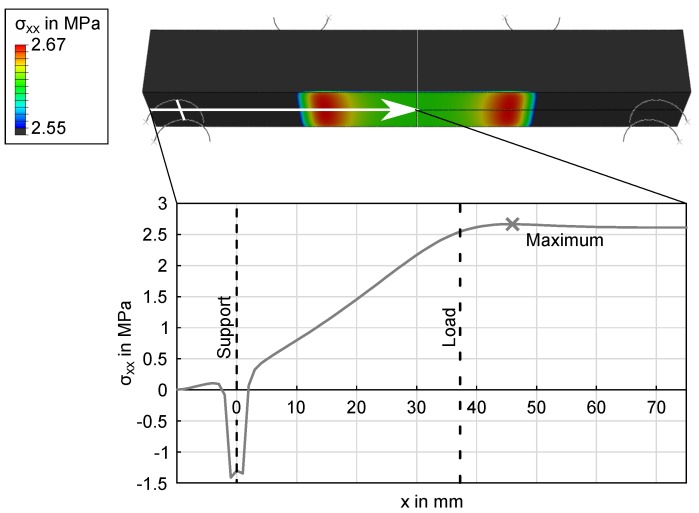
FEM results show the stress in the *x*-direction of 4PB 75.5 mm with a refined scale (top) and the stress distribution along the lower surface of the bar (bottom).

**Figure 12:**
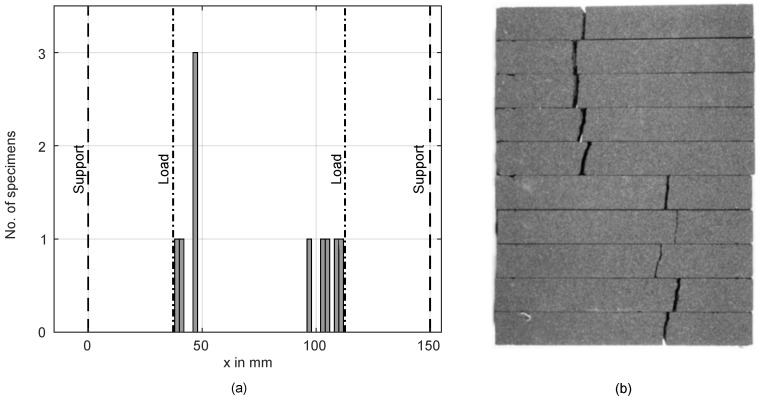
Fracture locations for a 4PB experiment with a 75.5-mm load distance (**a**) and the broken bending beams (**b**).

In order to investigate the observed overestimation, FEM calculations were performed to determine modeling errors induced by the ideal beam theory calculations. The FEM models described in Section Section 2.3 were utilized to calculate stresses for a three-point-bending load and four-point-bending loads with a 75.5-mm, 63-mm and 28-mm load distance. The results for the 3PB and the 4PB with a 75.5-mm load distance are shown in Figure 10 and Figure 11. While beam theory predictes a constant moment between the load points in a 4PB load case, the FEM simulation shows symmetrical stress concentrations 8.75 mm from the load points for a 75.5-mm load distance. To validate these results, another four-point-bending experiment was performed. Ten specimens of Type C were tested, and the location of the crack was measured on the tensile side of the beam, relative to the coordinate system in Figure 1. The results are shown in Figure 12. The fracture locations are clearly grouped around the stress maxima shown in Figure 11, supporting the results of the FEM simulation.

**Figure 13:**
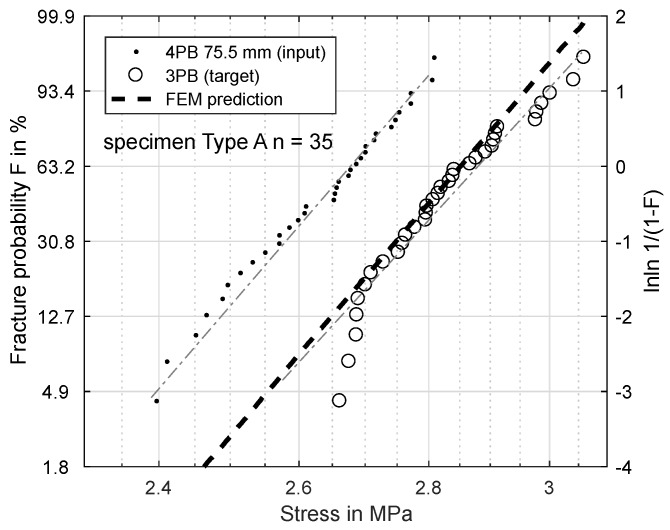
Specimen Type A: Prediction of fracture probability from 4PB (75.5 mm) to 3PB based on FEM analysis.

**Figure 14:**
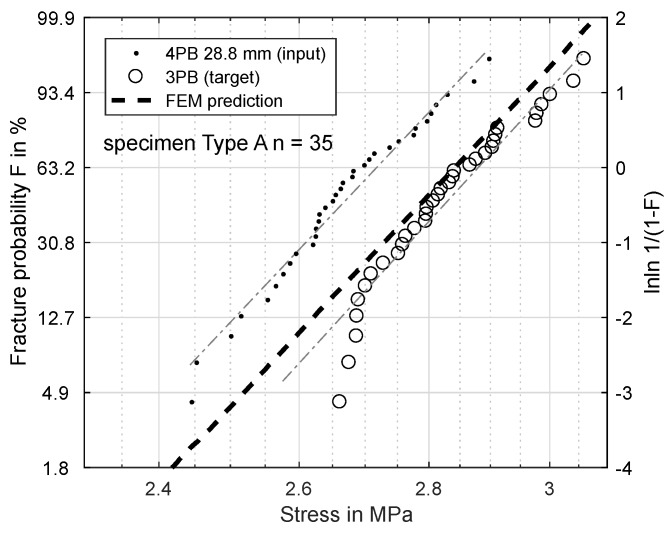
Specimen Type A: Prediction of fracture probability from 4PB (28.8 mm) to 3PB based on FEM analysis.

Based on these FEM results, the Weibull parameters from Figure 6, Figure 7, Figure 8 and Figure 9 were reevaluated with new effective volumes. Instead of the analytically-calculated stresses, the $\sigma_{xx}$ stresses from the simulations were numerically-integrated with the trapezoidal method [21] according to Equation (3) to obtain the specific effective volume. Figure 13 and Figure 14 show the results for the new prediction of the three-point-bending scale parameter $\sigma_{s}$ based on four-point-bending load cases with a 75.5-mm and 28.8-mm load distance. The scale parameter of the 3PB load case is predicted to be 2.851 MPa (4PB 75.5 mm) and 2.841 MPa (4PB 28.8 mm), compared to the measured scale parameter of 2.880 MPa. The reevaluation of Figure 8 and Figure 9 leads to predicted scale parameters of 2.738 MPa (measured: 2.717 MPa) and 2.854 MPa (measured: 2.901 MPa).

## 4. Discussion

### 4.1. Model Offset

There are two possible explanations for the observed overestimation of the fracture strength: First, the offset is some kind of measuring problem, resulting from the fixtures used. Two different fixtures were used for the three-point-bending and four-point-bending experiments. This means that the offset would have to be caused in a similar way by both fixtures. Moreover, the prediction for the three-point-bending based on the four-point-bending experiment with 28.8 mm fits the experimental data much better than that with 75.5 mm. This is probably due to the smaller difference in effective volume.

This makes the second option the more probable choice: The stresses were calculated according to ideal beam theory equations (Equations (4) and (5)). In reality, the stress state under the load and support points is more complex than these ideal circumstances. Finite element calculations of the four-point-bending experiment show that there are local stress concentrations under the load points on the tensile side, which will affect the real effective volume. This leads to an overestimation of $\sigma_{s}$. Obviously, these effects are included in all the measurements presented, but they do have more impact in cases of smaller effective volume, since the proportion of unmodeled stresses is greater. This explains why reducing the effective volume is weighted disproportionately, since the local stress concentrations, which are not modeled, account for a larger part of the real effective volume. The ideal analytic evaluation can serve as an upper boundary for the specimens’ strength, which is shown by Figure 6, Figure 7, Figure 8 and Figure 9. The results in Figure 13 and Figure 14 show that these stresses can be modeled by calculating the effective volumes based on FEM results. Moreover, the remaining error is now less dependent on the difference in effective volume during the prediction, which shows that the FEM-based model is more stable. The remaining prediction errors are well below 2% of the original absolute measured values.

### 4.2. Distinguishing between Volume and Surface Failure

In the Weibull theory, one can distinguish between surface and volume defects. For surface defects, an equation is deduced analogously to Equation (3). Usually, the quality of the fits for the surface and volume model is compared, which subsequently leads to the dominant failure type. However, it is not possible to separate between surface and volume failure for bending tests, since the surface area changes proportionally with the volume of the beam. Only a small fraction of the beam volume is contributing to the effective volume and the fracture probability in any case. Analyzing Equation (3) shows that the calculation of the Weibull parameters and the fracture process is predominantly influenced by the surface-near volume with the highest bending stress.

This shows that, at least for bending stresses, the mechanical properties of sand core materials are mainly influenced by the sand layers near the surface. This is especially important for simulations of the porous micro-structure of sand core materials [16], since it answers the question about from which part of the non-homogeneous cores the samples for the testing of material parameters should be taken.

## 5. Conclusions

Today’s state of the art of inorganically-bound cores comprises different fracture data for different specimens and experiments such as four-point-bending, three-point-bending, pressure and Brazilian tests. The resulting fracture stresses of these experiments were not comparable until now, since the influences of the stress state and the geometry of the specimens were unknown. In this article, we have shown that the fracture of inorganically-bound core materials follows a probabilistic Weibull distribution and that the fracture strength is influenced by the volume of the test specimen. Taking the effective volumes into account offers the possibility to compare different test specimens in the future. A Weibullian model for the fracture stress was fitted to four-point-bending experiments with constant uni-axial stress in the beam. Based on these parameters, an upper boundary was predicted analytically for the three-point-bending strength. Furthermore, it was shown that a prediction based on an FEM analysis leads to a more accurate and more stable model. In future work, we will extend the experimental setup to multi-axial stress fields and implement the model in an FEM software to simulate more complex stress states and geometries. In particular, the question of how an equivalent stress can be calculated to represent these complex stress tensors has to be answered. This allows the implementation of a Weibull material model for de-coring simulations and to predict the fracture stress and location of the cast-in cores.

## Abbreviations

The following abbreviations are used in this manuscript:

3PB	3-point-bending
4PB	4-point-bending
FEM	Finite element method
SEM	Scanning electron microscope
